# Intra-articular Injection of Mesenchymal Stem Cells After High Tibial Osteotomy in Osteoarthritic Knee: Two-Year Follow-up of Randomized Control Trial

**DOI:** 10.1093/stcltm/szac023

**Published:** 2022-06-08

**Authors:** Jun-Ho Kim, Kang-Il Kim, Wan Keun Yoon, Sang-Jun Song, Wook Jin

**Affiliations:** Department of Orthopaedic Surgery, Center for Joint Diseases, Kyung Hee University Hospital at Gangdong, Seoul, Republic of Korea; Department of Orthopaedic Surgery, Center for Joint Diseases, Kyung Hee University Hospital at Gangdong, Seoul, Republic of Korea; Department of Orthopaedic Surgery, School of Medicine, Kyung Hee University, Seoul, Republic of Korea; Department of Orthopaedic Surgery, Center for Joint Diseases, Kyung Hee University Hospital at Gangdong, Seoul, Republic of Korea; Department of Orthopaedic Surgery, School of Medicine, Kyung Hee University, Seoul, Republic of Korea; Department of Radiology, Kyung Hee University Hospital at Gandong, Seoul, Republic of Korea

**Keywords:** adipose, derived mesenchymal stem cell, knee, varus malalignment, osteoarthritis, high tibial osteotomy, medial open, wedge high tibial osteotomy

## Abstract

Intra-articular injection of adipose-derived mesenchymal stem cell (ADMSC) after medial open-wedge high tibial osteotomy (MOWHTO) would be a promising disease-modifying treatment by correcting biomechanical and biochemical environment for arthritic knee with varus malalignment. However, there is a paucity of clinical evidence of the treatment. This randomized controlled trial (RCT) was aimed to assess regeneration of cartilage defect, functional improvement, and safety of intra-articular injection of ADMSCs after MOWHTO compared with MOWHTO alone for osteoarthritic knee with varus malalignment. This RCT allocated 26 patients into the MOWHTO with ADMSC-injection group (*n* = 13) and control (MOWHTO-alone) group (*n* = 13). The primary outcome was the serial changes of cartilage defect on periodic magnetic resonance imaging (MRI) evaluation using valid measurements until postoperative 24 months. Secondary outcomes were the 2-stage arthroscopic evaluation for macroscopic cartilage status and the postoperative functional improvements of patient-reported outcome measures until the latest follow-up. Furthermore, safety profiles after the treatment were evaluated. Cartilage regeneration on serial MRIs showed significantly better in the ADMSC group than in the control group. The arthroscopic assessment revealed that total cartilage regeneration was significantly better in the ADMSC group. Although it was not significant, functional improvements after the treatment showed a tendency to be greater in the ADMSC group than in the control group from 18 months after the treatment. No treatment-related adverse events, serious adverse events, and postoperative complications occurred in all cases. Concomitant intra-articular injection of ADMSCs with MOWHTO had advantages over MOWHTO alone in terms of cartilage regeneration with safety at 2-year follow-up, suggesting potential disease-modifying treatment for knee OA with varus malalignment.

## Graphical Abstract

**Graphical Abstract:**
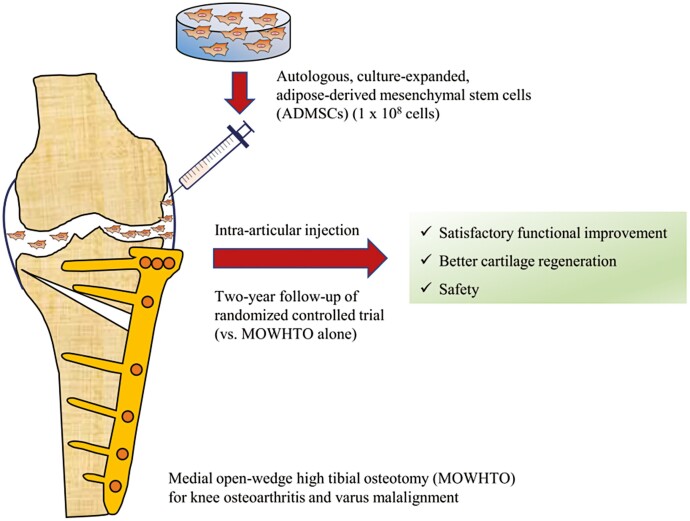

**Lessons Learned**

- Intra-articular injection of autologous, culture-expanded, adipose-derived mesenchymal stem cells after medial open-wedge high tibial osteotomy for knee osteoarthritis with varus malalignment provided functional improvements and significant cartilage regeneration with safety until 2-year follow-up.
- Results suggest that the treatment can be considered as a promising disease-modifying modality for knee osteoarthritis by means of correcting biomechanical and biochemical environment of osteoarthritic knee.

**Significance Statement**

This study was a prospective, randomized, open-label, blind endpoint, and control trial in patients with knee osteoarthritis and varus malalignment. An intra-articular injection of the autologous, culture-expanded, adipose-derived mesenchymal stem cells after high tibial osteotomy provided satisfactory functional improvement and better cartilage regeneration compared with high tibial osteotomy alone, confirmed by serial magnetic resonance imaging evaluations during 2-year follow-up without any safety issue. The treatment can be considered as a promising disease-modifying modality for knee osteoarthritis with varus malalignment by correcting biomechanical and biochemical environment of the knee.

## Introduction

Knee osteoarthritis (OA) is a degenerative and inflammatory joint disorder, affecting approximately 650 million global population of age over 40 years in 2020.^1-3^ As knee OA is a chronic progressive condition, it ultimately results in persistent knee pain, deformity, disability, and economic impacts for patients.^2,3^ However, current treatments have little impact on viable disease-modifying therapies for knee OA.^2,4^ Hence, patients with intractable symptoms and advanced stages of OA eventually undergo joint replacement surgery; however, several concerns about the surgery exist regarding patients’ comorbidity, limited motion, decreased function, complications, and short longevity of the implant.^5-9^ In this regard, developing effective and viable disease-modifying treatment is now considered to be a medical priority for knee OA.^10,11^

The primary pathogenesis of knee OA involves alteration of the biomechanical and biochemical environment in the joint which leads to a destructive process in cartilage.^2,10,12^ Among biomechanical environments, varus malalignment is the most common deformity and is highly associated with progressing medial compartmental OA, because it potentially induces excessive contact pressure on the medial side of the varus knee which accelerates wear and degradation of articular cartilage.^13,14^ Therefore, realignment surgery such as medial open-wedge high-tibial-osteotomy(MOWHTO) has been introduced to provide a favorable biomechanical environment by lateral shifting of weight load and showed effective clinical results to postpone joint replacement surgery.^15-17^ Furthermore, various degree of cartilage regeneration on the medial compartment has been also reported after MOWHTO in the literature although it is still debatable.^16,18,19^ However, MOWHTO itself would not be an ideal answer to the disease-modifying treatment for knee OA because the biochemical environment cannot be fundamentally changed.

Recently, mesenchymal stem cell(MSC)-based therapies have emerged as a promising regenerative medicine and have been increasingly investigated to modify the biochemical environment of the arthritic knee owing to its ability for chondrogenic differentiation and immune-modulatory properties; which may skew the biochemical environment of OA into regenerative and anti-inflammatory condition.^10,20,21^ In this context, recent meta-analyses with randomized-control trials (RCTs) have shown that intra-articular injection of MSCs led to significant pain relief and functional improvement with safety in patients with knee OA after the injection.^22-24^ However, the obvious efficacy of MSCs on articular cartilage regeneration remains still unclear.^22-24^

With a desire to challenge the nature of knee OA, intra-articular MSC-based therapy with MOWHTO in varus knee has been attempted lately, given the efficacy of regenerative medicine would be enhanced when coupled with biomechanical correction by the osteotomy.^10,12^ Although several studies have shown the efficacy of MOWHTO concomitant with intra-articular injection of MSCs,^25-27^ as for the RCT study, only one with bone marrow-derived MSCs exists with some limitations.^26^ The trial had performed MOWHTO with additional cartilage repair procedure(microfracture) and evaluated a single magnetic resonance imaging(MRI) assessment at postoperative 1 year without preoperative MRI, which might be critical confound factors for assessing articular cartilage changes.^26^ We designed an RCT to investigate the pure efficacy and safety of concomitant intra-articular injection of MSCs with MOWHTO for knee OA. Among various sources of MSCs, autologous adipose tissue has become an attractive option due to its easy accessibility, abundance, and safety.^23,28^ Furthermore, adipose-derived mesenchymal-stem-cells (ADMSCs) are theoretically assumed to have higher potential efficacy than adipose-derived stromal-vascular-fractions(ADSVF) because they are cultured for cell expansion and consist of homogenous MSCs.^29,30^ Nevertheless, insufficient information regarding the role of intra-articular injection of ADMSCs in knee osteoarthritis may lead some clinicians to conclude that it is not appropriate for the management of osteoarthritis.^31^ With this viewpoint, we performed an MSC-based therapy with autologous ADMSCs after MOWHTO without additional cartilage repair procedures to avoid possible confounding factors.

The purpose of our RCT was to evaluate the articular cartilage regeneration, clinical improvements, and safety of a single intra-articular injection of autologous ADMSCs after MOWHTO compared to MOWHTO alone until 2-year follow-up. We hypothesized that patients receiving an intra-articular injection of autologous ADMSCs after MOWHTO would show better articular cartilage regeneration and greater clinical improvements in safety than MOWHTO alone.

## Methods

### Study Design and Patient Selection

This prospective, randomized, open-label, blind end-point (PROBE), 2-arm parallel, controlled trial was conducted at a single institution. The trial was approved by the institutional review board of the institution (KHNMC2016-03-001-050) and registered at www.ClinicalTrials.gov (NCT03000712) prior to the enrollment of the first patient. Written informed consent was obtained from all participants.

**Figure 1.**
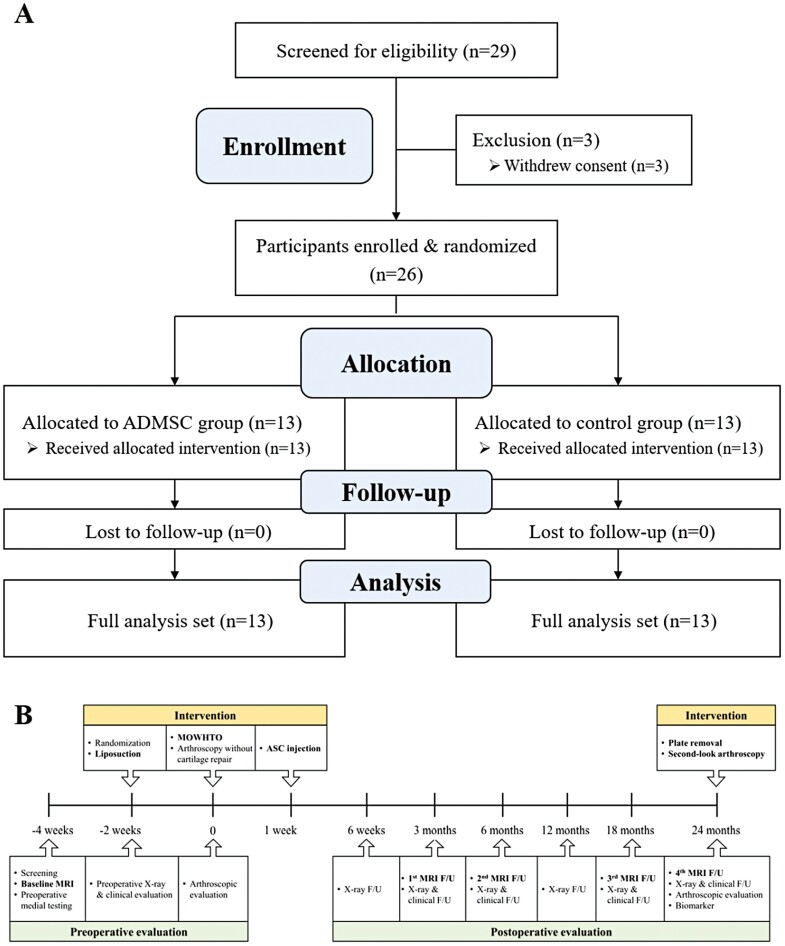
Consolidated standards of reporting trials (CONSORT) flow diagram (**A**) and detailed study protocol (**B**). After screening 29 patients, 26 patients were finally enrolled and randomized to ADMSC group (*n* = 13) or control group (*n* = 13). All patients completed a 2-year follow-up; thus full analysis set was performed in this RCT (A). For the ADMSC group, liposuction was conducted 2 weeks before MOWHTO and intraarticular injection of ADMSCs was performed 1 week after MOWHTO under ultrasound guidance. Serial MRI evaluations were performed at postoperative 3, 6, 18, and 24 months and two-stage arthroscopic evaluations of cartilage status were performed at the time of MOWHTO and at the time of plate removal at postoperative 24 months (B). Abbreviations: ADMSC, adipose-derived mesenchymal stem cell; MOWHTO, medial open-wedge high tibial osteotomy; MRI, magnetic resonance imaging; RCT, randomized controlled trial.

Eligible patients were aged 20-80 years and had symptomatic medial compartment knee OA (Kellgren-Lawrence[K-L] grades 2-4) assessed according to the American College of Rheumatology criteria^32^ with varus malalignment more than 5 degrees. The inclusion and exclusion criteria are detailed in Supplementary Table 1. Twenty-nine patients were initially screened for eligibility and 3 patients were excluded due to withdrawal of consent before the allocation. From November 2016 to February 2018, 26 patients (26 knees) were enrolled in this prospective RCT (Fig. 1A).

### Randomization and Study Protocol

Following their enrollment in the study, 26 patients were randomized to undergo MOWHTO and concomitant intra-articular injection of ADMSCs (ADMSC group) or MOWHTO alone(control group) in a 1:1 ratio according to a randomization schedule that was based on a randomized permuted block design with a block size of 4-6 (Fig. 1A).^33^ After the assessment of eligibility by the clinician, the research coordinator introduced and explained the study to the patients, using a standardized script. Patients who met the inclusion and exclusion criteria to participate in the study were assigned a randomized ID number and were allocated to either the ADMSC group or the control group.

Eligible patients underwent physical examination, laboratory tests, pregnancy test if needed, and MRI of the knee at the time of screening after informed consent. Lipoaspiration was performed 2 weeks before MOWHTO. A two-stage arthroscopic evaluation was performed at the time of MOWHTO and the time of plate removal at postoperative 24 months (Fig. 1B).

### ADMSC Preparation and Intervention

ADMSCs were isolated from abdominal subcutaneous fat by lipoaspiration and cultured under Good Manufacturing Practices conditions.^34,35^ Lipoaspiration was performed using the tumescent technique with 3-5 cc infiltration per 1 cc aspiration.^36^ The procedure of aspiration of adipose tissue was performed as followings: (1) aseptic skin preparation; (2) local anesthesia using 2% lidocaine; (3) 2 mm of stab incision using scalpel blade no. 11; (4) infiltration of the tumescent solution into the subcutaneous fat layer of the harvest site; (5) approximately 20 mL of adipose tissue was obtained through a metal cannula 10-15 min after the infiltration; (6) the obtained adipose tissue with the tumescent solution was carried at 2°C-8°C aseptic package and delivered to the laboratory; and (7) skin closure with 5-0 nylon (Ethicon, Somerville, New Jersey, USA). The aspirated adipose tissues were digested with collagenase I (1 mg/mL) under gentle agitation for 60 min at 37°C. To extract cellular debris, the digested tissues were filtered through a 100-µm nylon sieve and centrifuged at 470*g* for 5 min to collect a pellet. The pellet was resuspended in Dulbecco’s modified Eagle’s medium (Invitrogen, USA)-based media containing 0.2 mM ascorbic acid and 10% fetal bovine serum (FBS). The cell suspension was recentrifuged at 470*g* for 5 min. The supernatant was discarded and the pellet was obtained. The cell fraction was cultured for 4-5 days in Keratinocyte-SFM (Invitrogen, USA)-based media containing 0.2 mM ascorbic acid, 0.09 mM calcium, 5 ng/mL recombinant epidermal growth factor (rEGF), and 5% FBS until confluent (passage 0). When the cells reached 90% confluence, they were subculture-expanded in keratinocyte-SFM-based media containing 0.2 mM ascorbic acid, 0.09 mM calcium, 5 ng/mL rEGF, and 5% FBS until passage 3. The aliquots of culture-expanded MSCs were then tested for cell number, viability, purity (CD31, CD34, CD45), identity (CD73, CD90), and sterility including fungal, bacterial, endotoxin, and mycoplasma contamination as demanded by the Code of Federal Regulations, Title 21(21 CFR) before shipping. The culture-expanded MSCs maintained a survival rate of more than 80% for 72 h at 2°C-8°C. This high purity was shown by the persistent expression of surface antigen for MSCs up to 72 h. The cells were made and shipped on the day of injection.

The intra-articular injection was performed by a single investigator (RS) under the ultrasound guidance at *1 week after MOWHTO in the ADMSC group and 1 × 10^8^ cells of MSCs in 3 mL of normal saline was administrated. The dose of cells for intra-articular injection was determined by the result of the previous studies,^34,37^ because previous studies showed that intra-articular injection of high-dose (1 × 10^8^) ADMSCs in the knee OA improved pain and function without increasing AEs, and reduced cartilage defects by regeneration of hyaline-like articular cartilage, compared to low-dose (1 × 10^7^) and mid-dose (5 × 10^7^) ADMSCs.^37,38^ After the injection, patients were instructed to limit the use of the affected leg for at least 24 h.

### Surgical Technique and Rehabilitation Protocol

All patients were operated on by a single senior surgeon (KIK) and received arthroscopic examination during the MOWHTO to evaluate cartilage status as well as other intra-articular structures. No additional cartilage repair procedures such as chondroplasty or microfracture were performed. As a detailed technique was previously described,^18,39^ bi-planar MOWHTO was performed using a minimally invasive technique and fixed using a medial locked plate system (TomoFix; Synthes; Solothurn, Switzerland) after the osteotomy.^40,41^

Passive and active range of motion (ROM), quadriceps setting, straight-leg raises, and ankle pump exercises were started on the day after surgery. Partial weight-bearing ambulation with crutches was initiated when the pain was tolerable. Patients were permitted to begin full weight-bearing without crutches at 6 weeks after the surgery. Patients were also allowed to play tennis, cycle, or climb based on their demand and physical condition after the surgery. All patients followed the same protocol.

### Primary Outcome

The primary outcome was the changes in the area of articular chondral defect on serial MRI evaluations through postoperative 3, 6, 18, and 24 months from baseline MRI evaluation. MRI was performed using 3.0-T MRI (Philips, Amsterdam, the Netherlands) and all patients were performed with the same MRI protocol at all time periods. The assessment of articular cartilage defect was performed on the 3.0-mm T_2_-weighted Dixon in-phase sequence in sagittal and coronal images. To assess the changes in the cartilage defect area of medial femoral condyle on MRI, 3 widely used methods for cartilage evaluation were carried out as follows: (1) calculating the area of the regenerated articular cartilage on MRI from the change in the area of cartilage defect^34,37,42^; (2) Magnetic-Resonance-Observation of Cartilage-Repair-Tissue (MOCART) 2.0 knee score^43^; and (3) articular cartilage grading system of MRI-Osteoarthritis-Knee-Scores (MOAKS).^44^ Calculating area of the articular cartilage defect was measured by multiplying the anteroposterior (sagittal plane) and the mediolateral (coronal plane) diameter, which was defined as the maximum diameter of the articular cartilage defect with grades 3 or 4 of the modified Outerbridge grading system^45^ in the medial compartment.^34,37^ To assess the regeneration of articular cartilage, the proportion of change in cartilage defect area was calculated as (1 – postoperative defect area/baseline defect area) and compared the proportion between the ADMSC group and the control group.^34,37^ On serial follow-up MRIs, the cartilage regeneration tissue was also evaluated using MOCART 2.0 knee scores, of which 100 was the best possible score, and 0 was the worst possible score. Then we compared the MOCART scores between 2 groups.^43^ The rationale for the articular cartilage score of MOAKS was to provide separate scores for the size and depths of cartilage defect, which was also compared between the 2 groups.^44^ To reinforce the reliability of this open-label RCT, a PROBE design was conducted in this study. All of the radiological evaluations were performed by 2 independent radiologists (W.J. and J.H.K.) in a blinded manner.

In addition, subgroup analysis in the ADMSCs group was performed to assess the relationship between cell surface markers and the degree of cartilage regeneration on serial MRI.

### Secondary Outcomes

#### Macroscopic Cartilage Assessment

For the macroscopic assessment of articular cartilage status, a 2-stage arthroscopic examination was performed at the time of MOWHTO and plate removal at postoperative 2 years. Through the 2-stage arthroscopy, the articular cartilage defect was recorded according to the International Cartilage Repair Society(ICRS) grade,^46^ and the grade of cartilage regeneration after MOWHTO was classified based on the macroscopic staging system by Koshino et al,^19^ described as follows: stage A, no regeneration; stage B, partial regeneration (such as pink fibrous tissue with or without partial coverage with white fibrocartilage); and stage C, total regeneration. The grade of articular cartilage regeneration at 2 years after MOWHTO was compared between the ADMSC group and the control group. To decrease potential bias, interpretation of cartilage regeneration through 2-stage arthroscopy was performed by 2 independent physicians (J.H.K. and W.K.Y) in a blind manner.

#### Clinical and Radiologic Assessments

The postoperative improvements in functional patient-reported outcome measures (PROMs) including Western-Ontario and McMaster-Universities-Osteoarthritis-Index (WOMAC)^47^ and Knee-Injury and Osteoarthritis-Outcome-Score (KOOS)^48^ were assessed at serial postoperative 3, 6, 18, and 24 months from preoperative PROMs. In addition, preoperative and postoperative ROM were evaluated. All the clinical evaluations were compared between 2 groups and performed by an independent blinded physician, and the clinical research coordinator was blinded to the treatment as well. Radiological outcomes included the K-L grade, hip-knee-ankle angle,^49^ medial-proximal-tibial angle,^50^ posterior-tibial-slope angle,^51,52^ and correction angle during MOWHTO. Those variables were compared between 2 groups.

#### Biomarker Assessment

Serum biomarkers such as cartilage oligomeric matrix protein (COMP), C-terminal telopeptide of collagen type-I (CTX-I), C-terminal telopeptide of collagen type-II (CTX-II), Interleukin-10 (IL-10), tumor necrosis factor-inducible gene-6 (TSG-6), and urine biomarker of CTX-II were analyzed using enzyme-linked immunosorbent assay (ELISA) at postoperative 24 months. Aspirated synovial fluid was also analyzed using ELISA for thrombospondin-2 (TSP-2) at the time of plate removal at 24 months follow-up.

### Safety and Complications

Safety was assessed with adverse events (AEs), serious adverse events (SAEs), vital signs, physical examination, electrocardiogram, and laboratory tests. The severity of AEs was determined based on the National Cancer Institute-Common Terminology Criteria for Adverse Events(NCI-CTCAE).^53^ Causality assessment for AEs caused by the intervention was determined and recorded according to the World Health Organization-Uppsala Monitoring Centre causality assessment system when AEs occurred.^54^ Postoperative complications were also reviewed including symptomatic deep vein thrombosis, wound dehiscence, infection, and failures such as conversion to arthroplasty or reoperation.

### Statistical Analysis

#### Sample Size Calculation

A priori sample size determination was based on the prior study^37^ to detect a 200.1 mm^2^ difference in the articular cartilage defect area, a 2-tailed test, an SD of 189.64 mm,^2^ an *α* value of 0.1, and a power (*β*) of 0.8, resulting in 12 participants per group. To account for possible losses to follow up, a loss rate of 7% was assumed, and an additional one participant per group was added, thus we decided to recruit 13 participants in each group.

#### Statistics

In the present study, statistical analyses were performed on the full analysis data set. The Kolmogorov-Smirnov test was applied to the continuous data to determine if they follow a normal distribution. Baseline demographic characteristics and the mean improvement from baseline in each clinical outcome at each follow-up visit were assessed for each patient. The 2 study cohorts were compared using Student’s t test, the Mann-Whitney *U test*, or the Pearson chi-square test. For subgroup analysis in the ADMSC group, a simple linear regression analysis was performed to assess whether any of cell surface markers (CD31, CD34, CD45, CD73, and CD90) used in the current study has an association with any of the 3 methods on MRI regarding cartilage regeneration. Data were analyzed using SPSS software (version 21.0; IBM Corp., IL, USA) and R statistical software (version 4.0.2). *P*-values were adjusted for multiple comparisons using the Benjamini-Hochberg procedure, and the false discovery rate (FDR) adjusted *P*-value was calculated.^55^ Two-way FDR-adjusted *P*-values lower than .05 were assumed to be statistically significant.^55^

To identify the reliability and reproducibility of arthroscopic findings, intra- and inter-observer errors were evaluated using the intra-class correlation coefficient (ICC) method, and ICC was classified as little if any values were ≤0.25, low if 0.26-0.49, moderate if 0.50-0.69, high if 0.70-0.89, or very high if ≥0.90.^56^

## Results

All patients completed a 2-year follow-up (Fig. 1A). Only one patient in the control group refused to take MRI at postoperative 24 months; however, the patient completed other evaluations. Meanwhile, the 2 groups showed no significant difference in patients’ demographics, K-L grade for OA, radiologic variables of alignments, correction angle during MOWHTO, and cartilage status based on ICRS grade of arthroscopic findings at baseline (Table 1).

**Table 1.** Patients’ demographics and radiologic characteristics.

	ADMSC (*n* = 13)	Control (*n* = 13)	*P* value
Age, years	58.3 ± 6.4	59.1 ± 5.9	.754^a^
Sex, female/male, *n*	11/2	8/5	.378^d^
Operated side, right/left, *n*	8/5	7/6	>.999^c^
BMI, kg/m^2^	25.6 ± 2.7	25.8 ± 2.6	.887^a^
Smoking status, *n*			>.999^d^
Never smoked	11	11	
Current smoker	2	2	
Comorbidities, *n*			.883^c^
None	4	5	
HTN/DM/dyslipidemia	7/3/1	6/1/2	
Range of motion, °			
Preoperative	135.8 ± 6.1	136.2 ± 8.2	.893^a^
Postoperative, 2 years	145.0 ± 5.8	145.0 ± 4.1	>.999^a^
K-L grade, *n*			.688^c^
Grade 2/3/4	4/9/0	6/7/0	
HKAA^†^, °			
Preoperative,	−6.6 ± 1.4	−6.7 ± 1.9	.833^a^
Postoperative, 2 years	3.0 ± 1.4	2.2 ± 1.4	.155^a^
MPTA, °			
Preoperative,	83.6 ± 1.2	83.5 ± 1.4	.840^a^
Postoperative, 2 years	93.4 ± 2.3	92.9 ± 1.6	.545^a^
Posterior slope, °			
Preoperative,	8.7 ± 1.6	7.2 ± 2.4	.090^a^
Postoperative, 2 years	9.5 ± 2.8	9.2 ± 3.2	.785^a^
Correction angle, °	9.9 ± 1.4	9.6 ± 1.5	.601^a^
Baseline Intraoperative ICRS grade, *n*			
MFC, 3/ 4	3/ 10	4/ 9	>.999^c^
MTP, 2/ 3/ 4	1/ 7/ 5	1/ 4/ 8	.695^c^

Values are present as mean ± SD.

Student *t*-test.

Mann-Whitney *U test*.

Pearson chi-square test.

Fisher’s exact test.

Statistical significance was set at <.05.

Abbreviations: ADMSC, adipose-derived mesenchymal stem cell; BMI, body mass index; DM, diabetes mellitus; FC, flexion contracture; FF, further flexion; HKAA, hip-knee-ankle angle; HTN, hypertension; MFC, medial femoral condyle; MPTA, medial proximal tibial angle; MTP, medial tibial plateau; ICRS, International Cartilage Repair Society; K-L, Kellgren-Lawrence.

### Primary Outcome

The proportion of the regenerated articular cartilage area was significantly greater in the ADMSC group than in the control group during all follow-up periods after the treatment (Table 2). The mean MOCART score was significantly higher in the ADMSC group than in the control group during most follow-up periods (Table 2). The cartilage defect of MOAK grade showed no significant difference between the 2 groups at baseline. Meanwhile, the MOAK grade of the ADMSC group showed significantly smaller the size of cartilage defects than those of the control group at postoperative 24 months in sagittal (full-thickness cartilage defect; *P* = .043) and coronal planes (any cartilage defect; *P* = .015) (Supplementary Fig. S1). In subgroup analysis in the ADMSC group, there was no significant relationship between cell surface markers and the degree of cartilage regeneration on serial MRI (Supplementary Table S2). The ICCs for chondral defect area, MOCART score, and MOAK grade were between 0.76 and 0.88, 0.84 and 0.95, and 0.88 and 0.96, respectively, indicating high intra-observer and inter-observer agreement.

**Table 2.** Changes in cartilage from baseline to 24 months based on cartilage defect area and MOCART 2.0 knee scores using MRI.

	ADMSC (*n* = 13)	Control (*n* = 13)	95% CI	*P* value	*P* value^†^
Mean cartilage defect area, mm^2^					
Baseline	205.0 ± 181.4	296.1 ± 203.0	−246.9 to 64.8	.240^a^	.240^a^
3 months	137.4 ± 171.7	246.3 ± 192.	−257.8 to 40.0	.144^a^	.201
6 months	106.89 ± 175.4	226.5 ± 176.3	−261.9 to 22.7	.096^a^	.201
18 months	90.4 ± 186.6	197.3 ± 172.9	−252.6 to 38.7	.143^a^	.201
24 months	81.5 ± 186.1	178.9 ± 155.6	−236.2 to 41.5	.161^a^	.201
Ratio of regenerated cartilage area, %					
3 months/baseline	43.3 ± 30.1	17.4 ± 20.9	4.9 to 46.9	.018^a^^,^^*^	.031^*^
6 months/baseline	65.3 ± 40.1	27.4 ± 36.7	6.7 to 69.0	.019^a^^,^^*^	.031^*^
18 months/baseline	74.9 ± 37.9	38.5 ± 43.2	3.6 to 69.4	.031^a^^,^^*^	.031^*^
24 months/baseline	81.1 ± 34.4	44.4 ± 43.8	4.8 to 68.6	.026^*^	.031^*^
MOCART 2.0 knee score					
3 months	40.0 ± 20.1	28.1 ± 13.6	−2.1 to 25.9	.091^a^	.091
6 months	58.5 ± 26.3	33.8 ± 22.4	4.8 to 44.4	.017^a^^,^^*^	.034^*^
18 months	64.6 ± 27.5	40.4 ± 24.5	3.1 to 45.3	.026^a^^,^^*^	.034^*^
24 months	76.2 ± 23.6	50.4 ± 28.9	4.4 to 47.1	.020^a^^,^^*^	.034^*^

Values are present as mean ± SD.

Student *t*-test.

Mann-Whitney *U* test.

*P*-value was adjusted for multiple comparisons using the false discovery rate (FDR).

Statistical significance was set at <.05.

Abbreviations: ADMSC, adipose-derived mesenchymal stem cell; MOCART, magnetic resonance observation of cartilage repair tissue; MRI, magnetic resonance imaging.

### Secondary Outcomes

#### Macroscopic Findings

**Figure 2.**
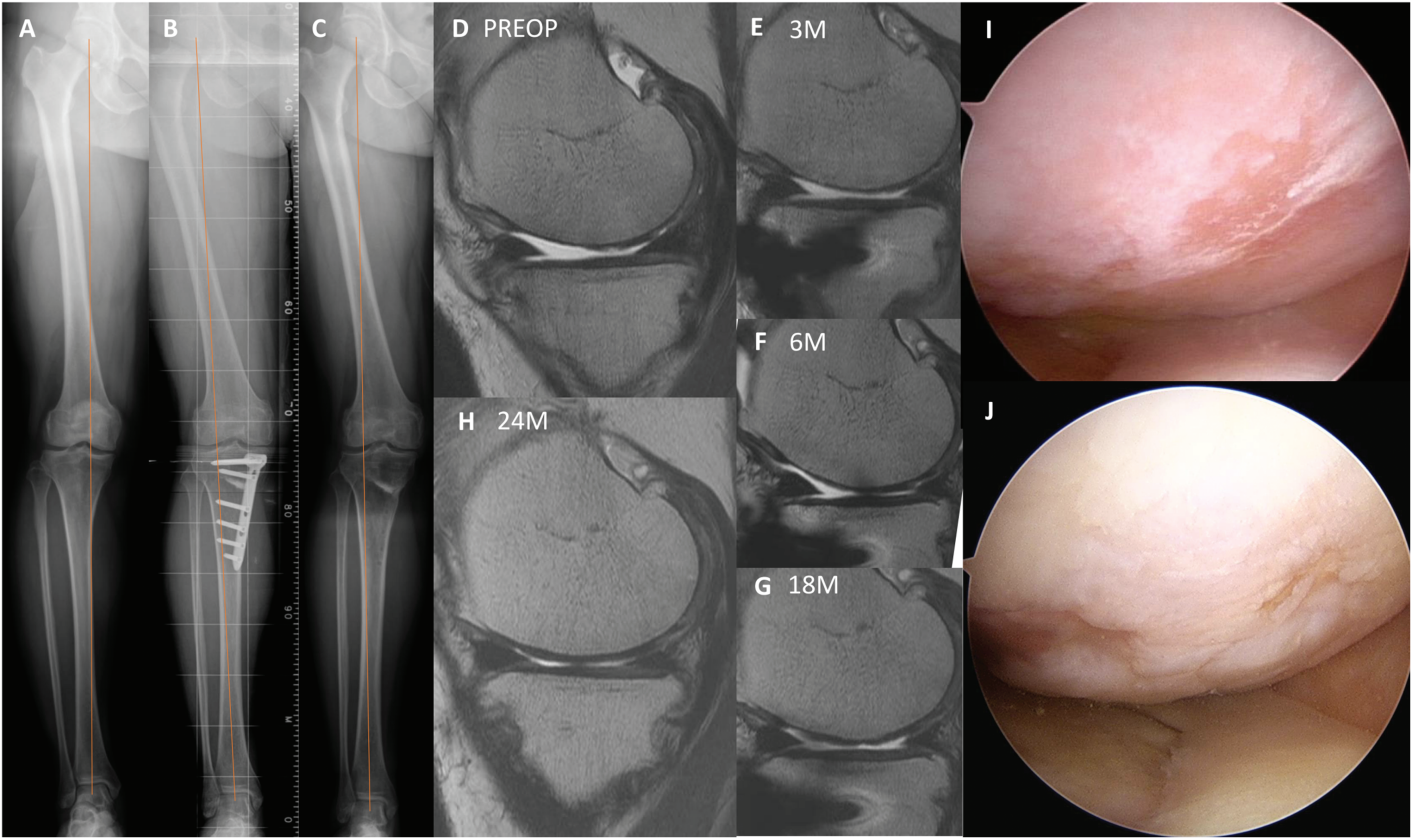
Representative case of MRI and arthroscopic changes after MOWHTO with intra-articular injection of ADMSCs. Preoperative medial compartment OA with varus alignment (A) of the right knee of a 50-year-old female patient is corrected to valgus alignment via MOWHTO (B) and maintained well at postoperative 2 years (C). Preoperative T2-weight sagittal image of MRI shows a full-thickness defect of articular cartilage in the MFC (D). Cartilage regeneration along favorable integration with adjacent native cartilage is observed through serial MRI follow-up up to postoperative 24 months (E-H). Arthroscopic findings of cartilage status in MFC show the exposure of subchondral bone at the time of initial arthroscopy (I) and nearly total coverage of regenerated cartilage 2 years after intra-articular injection of ADMSCs to MOWHTO (J). Abbreviations: ADMSC, adipose-derived mesenchymal stem cell; MFC, medial femoral condyle; MOWHTO, medial open-wedge high tibial osteotomy; MRI, magnetic resonance imaging; OA, osteoarthritis.

The degree of articular cartilage regeneration showed significantly better in the ADMSC group (total regeneration, 69.2%) than in the control group (total regeneration, 23.1%; *P* =.042) by macroscopic staging assessment through 2-stage arthroscopy (Fig. 2; Table 3). The ICC for macroscopic findings was between 0.86 and 0.94, indicating significantly high intra-observer and inter-observer agreement.

**Table 3.** Stage of regeneration of articular cartilage based on 2 stage arthroscopic findings.

	ADMSC (*n* = 13)	Control (*n* = 13)	*P* value
Koshino’s macroscopic grade			.042^*^
A (no regeneration)	0	2 (15.4)	
B (partial regeneration)	4 (30.8)	8 (61.5)	
C (total regeneration)	9 (69.2)	3 (23.1)	

Values are presented as no. (%).

Statistical significance was set at *P <* .05.

Abbreviation: ADMSC, adipose-derived mesenchymal stem cell.

#### Clinical Improvements and Radiological Outcomes

**Figure 3.**
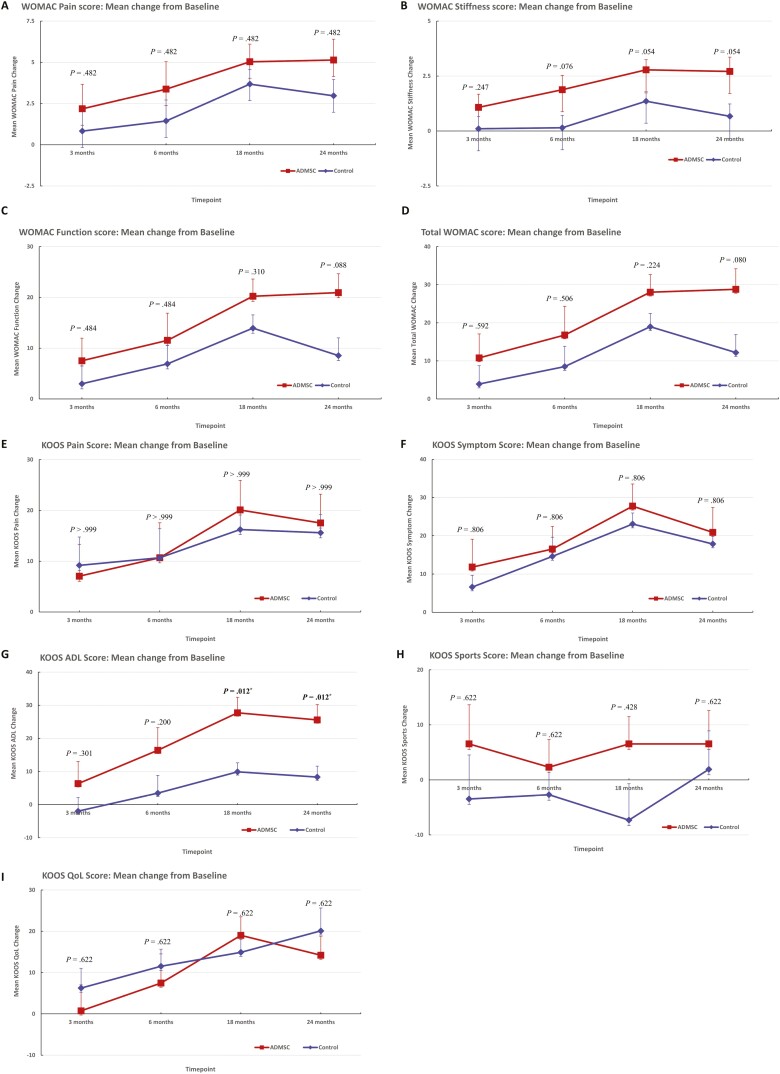
Comparison of mean improvement from baseline in WOMAC scores and KOOS at postoperative 3, 6, 18, and 24 months between intra-articular injection to MOWHTO (ADMSC group) and MOWHTO alone (control group). Patients in the ADMSC group had a tendency to show an improvement in WOMAC stiffness at postoperative 18 (*P* = .054) and 24 months (*P* = .054) compared to the control group (A, B). In addition, patients in the ADMSC group had a tendency to show an improvement in WOMAC function (*P* = .088) and total scores (*P* = .080) at postoperative 24 months compared to the control group (C, D). Patients in ADMSC group showed significantly higher improvement in KOOS ADL subscale at postoperative 18 (*P* = .012) and 24 (*P* = .012) months (E-I). Abbreviations: ADL, activities of daily living; ADMSC, adipose-derived mesenchymal stem cell; KOOS, knee injury and osteoarthritis outcome score; MOWHTO, medial open-wedge high tibial osteotomy; QoL, quality of life; WOMAC, Western Ontario and McMaster Universities Osteoarthritis Index.

Two groups showed no significant difference at baseline regarding WOMAC and KOOS scores (Supplementary Tables S3 and S4). Although it was not significantly different between the 2 groups regarding WOMAC scores (Fig. 3), the improvement of KOOS-ADL subscale was significantly greater in the ADMSC group than in the control group at 18 (*P* = .012) and 24 months (*P* = .012) (Supplementary Table S5; Fig. 3). Radiological variables and ROM at preoperative and postoperative outcomes showed no significant difference between the 2 groups.

#### Biomarker Outcomes

No significant difference was found between the 2 groups in serum, urinary, and synovial biomarkers at postoperative 24 months, although synovial TSP-2 tended to be higher in the ADMSC group (3.7 ± 1.7 ng/mL) than in the control group (1.5 ± 1.9 ng/mL) despite statistical insignificance (*P* = .09) (Supplementary Table S6).

### Safety and Complications

AEs occurred in 9 (69.2%) in each group and those were not treatment-related AEs. There were no grades 3, 4, or 5 AEs by the NCI-CTCAE scale and no SAEs (Table 4). No donor-site complication occurred in the ADMSC group. Details of AEs are summarized in Supplementary Table S7. There were no postoperative complications in both groups.

**Table 4.** Details of adverse events.

	ADMSC (*n* = 13)	Control (*n* = 13)
Patients with AEs, *n* (%)^a^	9 (69.2)	9 (69.2)
Treatment-related	0	0
Donor-site complication	0	-
Patients with SAEs, *n* (%)^b^	0	0
Treatment-related	0	0
AEs by NCI-CTCAE scale, *n*	13	21
Grade 1	8	6
Grade 2	5	15
Grade 3	0	0
Grade 4	0	0
Grade 5	0	0

An AE is defined as any undesired medical incident that does not necessarily have a cause-and-effect relationship with the treatment.

An SAE is defined as any undesired medical incident that causes death, life-threatening, hospitalization, disability, congenital abnormality, or birth-death.

Abbreviations: AE, adverse events; ADMSC, adipose-derived mesenchymal stem cell; CTCAE, National Cancer Institute-Common Terminology Criteria for Adverse Events; SAE, severe adverse events.

## Discussion

The main finding of this RCT was that intra-articular injection of autologous ADMSCs after MOWHTO showed better results than MOWHTO alone in OA knee with varus malalignment, with respect to cartilage regeneration and modest functional improvement without relevant AEs and complications until postoperative 2-year follow-up. Although few comparative studies have investigated cartilage regeneration and clinical efficacy of intra-articular injection of MSCs to MOWHTO,^12,25,26^ no RCT has been performed with serial MRI evaluations and assessment of macroscopic cartilage changes.

The primary etiology of knee OA is characterized by biomechanical and biochemical changes in the knee joint including the destructive course of articular cartilage.^2,10,12^ In a biomechanical aspect of the OA knee, since most knees with OA have various degrees of varus malalignment, excessive medial contact stress results in a meniscal degenerative tear and marked cartilage wear with varus progression which may induce incapability to tolerate excessive contact pressure and joint destruction.^13,14^ In the biochemical aspect, the following phenomenon can accelerate arthritic conditions such as a restricted supply of nutrients and oxygen, insufficient synthesis of extracellular matrix components, increased catabolic cytokines, apoptosis of chondrocytes, and synovial inflammation.^2,57^ Therefore, the ideal disease-modifying treatment for knee OA with varus malalignment should restore both biomechanical and biochemical environments in the joint to potentially regenerate articular cartilage and to improve functional outcomes. MOWHTO has been a well-established treatment to improve medial OA with the varus knee as it leads to decompression of excessive contact pressure on the medial side by shifting the load to the healthy lateral compartment through the valgus correction of the proximal tibia.^13,58^ Numerous studies have demonstrated that satisfactory functional improvements could be achieved after MOWHTO.^13,15,18^ Moreover, various degree of cartilage regeneration has been observed after MOWHTO irrespective of cartilage repair procedures; however, the quality and quantity of cartilage regeneration seemed to be still insufficient to guarantee the long-term outcomes.^16,18,59^ Meanwhile, MSC-based therapy may contribute to changing the biochemical environment because MSCs have known to not only differentiate into chondrocytes but also to have immune-modulatory and anti-inflammatory benefits through suppression of T-cell proliferation and monocyte maturation as well as expression of anti-inflammatory and anabolic cytokines.^10,20,60^ Many studies regarding the intra-articular MSC injection in OA knee have been reported with improved clinical outcomes and some degree of cartilage regeneration through MRI evaluation with safety, although the protocol of collagenase digestion and culture-expansion of MSCs is not still currently permitted in many countries.^10,22,60,61^ Moreover, Cho et al reported that intra-articular injection of autologous high-dose MSCs (1 × 10^8^) significantly decreased articular cartilage defect resulting from the regeneration of hyaline-like cartilage based on the histological evaluation.^37^ A case series reported that hyaline-like cartilage was also observed after concomitant intra-articular injection of MSCs with MOWHTO in arthritic varus knee.^27^ It may be postulated that the biochemical environment, as well as biomechanical environment, has been substantially improved to reflect the ameliorating function of the knee joint after the treatment.

In this regard, concomitant MSCs-based therapy with MOWHTO has recently emerged with the hope to improve the biochemical environment in addition to biomechanical correction.^12^ Several studies investigated the functional outcomes, cartilage regeneration, and safety of intra-articular injection of MSCs with MOWHTO.^12,25,26,62^ Since their study design was not that advanced, only one RCT has been reported among the studies.^26^ It found that the cell-recipient group (bone marrow-derived MSCs) showed significantly higher clinical improvement at postoperative 2 years and better cartilage regeneration in MOCART scores at postoperative 1 year with safety compared to MOWHTO alone.^26^ A recent meta-analysis involving 4 comparative studies also addressed that intra-articular injection of MSCs with MOWHTO may modestly improve functional outcomes as compared with MOWHTO alone.^12^ We designed an RCT to find out the efficacy and safety of intra-articular injection of autologous high-dose ADMSCs after MOWHTO through serial MRI evaluations until a 2-year follow-up. The current study found that intra-articular injection of ADMSCs with MOWHTO had shown significantly better cartilage regeneration from the postoperative 6 months as compared to MOWHTO alone. As the current study had a strength of serial MRI evaluations, we could provide more specific information on cartilage regeneration after MOWHTO with the injection of ADMSCs. Thin cartilage regeneration was noticed at 3 months and it became thicker and matured from 6 months after injection, which was consistent with previous studies.^37,38^ The maximal regeneration of cartilage on serial MRI was noted mostly at postoperative 2 years (76.9% in the ADMSC group and 69.2% in the control group). Interestingly, the signal intensity of regenerated cartilage represented slightly hypo-intensity, compared to normal cartilage, and maintained the signal till 2 years after the operation. It might be postulated that histology of regenerated cartilage showed a mixture of hyaline and fibrous cartilage after MOWHTO or intra-articular injection of MSCs, based on previous studies.^27,37,63^

Meanwhile, based on arthroscopic evaluation, total regeneration of articular cartilage was seen at 69.2% in the ADMSC group but 23.1% in the control group with a significant difference at postoperative 2 years. It is surprising results because recent RCTs were not able to draw consistent efficacy of cartilage regeneration after intra-articular injection of ADMSCs alone in knee OA despite their promising ability.^23,34,60,64,65^ It might be because the biomechanical environment has not changed and all of the follow-up periods were within 1 year after the treatment.^22,66,67^ Although it was expected that cartilage regeneration would be significantly improved when intra-articular injection of ADMSCs was coupled with MOWHTO for biomechanical correction, a recent meta-analysis was not able to make a definite conclusion for superior cartilage regeneration regarding intra-articular injection of MSCs in MOWHTO when compared with MOWHTO alone due to a lack of adequate data.^12^ Therefore, our result may contribute to the evidence of superior cartilage regeneration when biochemical correction was coupled with biomechanical correction in the arthritic knees. Taken together, we believe that intra-articular injection of autologous high-dose ADMSCs with MOWHTO seems an attractive and viable disease-modifying treatment for knee OA with varus malalignment, because it may challenge the degenerative course of OA in terms of enhancing cartilage regeneration by restoring biomechanical and biochemical environments of knee OA.

The present RCT revealed that patients in the ADMSC group had shown modestly greater functional improvements than patients in the control group from postoperative 18 months. Although it was not significantly different in WOMAC scores between the 2 groups, the improvement of WOMAC-stiffness, WOMAC-function, and WOMAC-total scores showed a tendency to have greater improvements in the ADMSC group than those in the control group, after the postoperative 18 months. Particularly, patients in the ADMSCs group had shown a significantly better improvement in KOOS-ADL scores than those in the control group from the postoperative 18 months. Because the MOWHTO itself has shown a substantial, satisfactory effectiveness on functional improvement in knee OA with varus malalignment,^13,15^ it would be difficult to achieve a statistically significant difference in functional improvements between 2 groups, owing to just a single intra-articular injection of ADMSCs. Nevertheless, a recent meta-analysis showed that intra-articular injection of MSCs with MOWHTO significantly improved functional outcomes as compared with MOWHTO alone,^12^ which was partially consistent with the result of the current study. However, the meta-analysis only included 4 studies with only one RCT^26^; 2 of included studies used uncultured-expansion MSCs^25,63^; one of the included studies was performed in 2002 without locked-plate fixation.^68^ In this regard, a lack of evidence remains on this topic. According to the result of the current RCT, functional improvements had shown a stronger tendency for greater improvements in the ADMCS group from the postoperative 18 months, as time spent, although statistical significance could not be fully achieved. Thus, we believe that a further RCT with a longer follow-up duration based on the current RCT would provide more robust information for intra-articular injection of MSCs with MOWHTO as an effective and viable therapeutic option for functional improvements in patients with knee OA.

Meanwhile, biomarkers in the present RCT were not able to reach a significant difference between the 2 groups although superior results in cartilage regeneration and functional improvement were observed in the ADMSC group. Recently, biomarkers have been used as a measure of the degree of OA processes and assessment of patients’ response to treatment, however, it has not been clearly defined which type of biomarkers ideally reflect the activity of OA.^69-71^ Both effector molecules, such as cytokines and growth factors, and extracellular matrix components, such as precursors or degradation products of collagen and proteoglycan, have been potentially used for biomarkers and their concentrations could be measured in serum, urine, or synovial fluid.^71^ Although we have used the widely used biomarkers such as COMP,^71^ the biochemical evidence from biomarkers could not be observed. It may be explained that most biomarkers used in the current study were measured from blood or urine, of which systemic biomarkers can reflect not only knee joints but also other degenerative joints resulting in confounding factors.^71^ In addition, the therapeutic efficacy of MSCs is considered to be mainly paracrine-mediated to deliver chondrogenic and immune-modulatory effects,^72^ thus local synovial biomarkers would be more appropriate to reflect the response to the MSC therapy than systemic biomarkers.^71^ Interestingly, synovial TSP-2, which was only included synovial marker in this study, tended to be higher in the ADMSC group (3.7 ± 1.7 ng/mL) than in the control group (1.5 ± 1.9 ng/mL) although there was no statistical significance (*P* = .09). TSP-2 is a known regulator of cartilage and is secreted by MSCs to promote cartilage regeneration showing evidence that TSP-2 is one of the main paracrine players in MSC-mediated cartilage regeneration.^72,73^ Despite the evidence that TSP-2 has been validated for its paracrine effect on chondrogenic differentiation in *in vitro* or *in vivo* animal models,^73,74^ a lack of data exists currently for the reference of TSP-2 in human studies. Therefore, it would be informative and interesting if our results contribute to following future studies to investigate similar research with synovial TSP-2 and would become the reference for synovial TSP-2 in human samples. Although the exact mechanism of ADMSCs for cartilage regeneration is difficult to know based on the result of the current study, it is prevailing speculation that paracrine action through the secretion of bioactive materials is a possible mechanism of the cartilage-restoring effect of ADMCSs, rather than the directly engraftment of injected ADMSCs, according to recent literature.^75,76^ Meanwhile, although the current study has limited evidence to show the biochemical evidence of ADMSCs by biomarkers, further studies with various synovial biomarkers and a larger sample size are required to demonstrate the clinical utility of biomarkers in MSC therapy for osteoarthritic knee.

While informative, some limitations of the current study need to be addressed. First, double-blinding was not conducted because sham procedures including invasive lipoaspiration and intra-articular injection of saline to the control group seemed ethically implausible to the operated patient despite valid strength. To decrease this limitation, we rather performed the PROBE design with external evaluators, who were blinded to the treatment allocation. Second, a 2-year follow-up period might be short to provide conclusive data for efficacy and safety regarding intra-articular injection of ADMSCs with MOWHTO. However, it would be the first RCT, as far as we know, to compare MOWHTO with ADMSCs injection and MOWHTO alone in OA patients with varus knee, thus 2-year results would be enough to provide meaningful information. Furthermore, we performed serial MRI evaluation for assessing cartilage changes with time. Third, because we evaluated Asian patients, the demographic characteristics of our trial population should be noticed before extrapolating our findings to other populations; more frequent varus malalignment and a marked female predominance in the knee OA population might be prominent differences to consider.^77^ Lastly, it would be more reliable and better if cartilage defects on MRI had been measured in 3-D shape with volumetric assessment. Unfortunately, it was not possible to measure chondral defects with volumetric measurement in this RCT. However, we believe that measuring chondral defects in 2-D shape is still a widely used, reliable, and valid assessment for cartilage change according to previous studies.^33,36,37,42^ Moreover, we performed 2 additional valid assessments of cartilage change using MOCART and MOAKS system.^43,44^

In conclusion, concomitant intra-articular injection of ADMSCs with MOWHTO had advantages over MOWHTO alone in terms of cartilage regeneration with safety at 2-year follow-up, suggesting potential disease-modifying treatment for knee OA with varus malalignment.

## Supplementary Material

szac023_suppl_Supplementary_Material

## Data Availability

The data that support the findings of this study are available on request from the corresponding author. The data are not publicly available due to privacy or ethical restrictions.
