# Supplementary material for: Intra-articular Injection of Mesenchymal Stem Cells After High Tibial Osteotomy in Osteoarthritic Knee: Two-Year Follow-up of Randomized Control Trial
Source: Stem Cells Transl Med. 2022 Jun 8;11(6):572–85. doi: 10.1093/stcltm/szac023 (PMC9216209; doi:10.1093/stcltm/szac023)
Supplement: szac023_suppl_Supplementary_Material [file szac023_suppl_supplementary_material.docx]

Supplementary Table 1. Inclusion and Exclusion Criteria

| Inclusion Criteria |
| --- |
| - Candidate for high tibial osteotomy: Symptomatic primary medial compartment osteoarthritis with varus malalignment (more than 5 degrees) needing to be corrected. - Age between 20 and 80 years old - Body mass index ≤ 30 kg/m^2^ - Kellgren-Lawrence grade 2 to 4 - Diagnosis of primary osteoarthritis of the knee, according to the American College of Rheumatology criteria - At least one focal or diffuse 3-4 International Cartilage Repair Society grade on baseline magnetic resonance imaging - Patient is able to understand the instruction by doctor and to complete the questionnaires required for the study. - Signing informed consent form |
| Exclusion Criteria |
| - Patients with measures twice or more than normal in lab test or with any condition that principle investigator considers clinically important. - Patients with serious condition which can affect this study such as cardiovascular diseases, renal diseases, liver diseases, endocrine diseases, cancer or diabetes. - Osteoporosis - Female who are pregnant, lactating, or planning pregnancy - Patients with positive at screening test of human immunodeficiency, hepatitis B or hepatitis C - Patients with history of hypersensitivity or allergy to any substances used within the treatments - Patients who had participated in other clinical trials within 12 weeks prior to this study. - Any knee intra-articular injection of any drug within 12 weeks before the screening - Patients with osteoarthritis who had any history of cartilage repair surgery or stem cell injection or implantation at affected knee - Patients who has changed or started established program of physical therapy before the screening. - Patients with other disease (no matter the length of time) including systemic or Rheumatoidal or inflammatory cartilage disease, crystalline disease (gout or pseudogout), hemochromatosis, inflammatory joint disease, femoral head necrosis, Paget disease in the joint of femur or tibia, or related knee joint disease, ochronosis, hemophilia arthropathy, joint infections, joint sarcoidosis, villonodular synovitis, or solitary synovial chondromatosis - History of liposarcoma or other cancer diagnosed within 5 years (included if patients had complete remission more than 3 years ago) - Patients who received contraindicated drugs without wash-out period for this study - Patients who the principal investigator considers inappropriate for the clinical trial due to any other reasons than those listed above (i.e. cognitive dysfunction, lack of will…) |

Supplementary Table 2. Simple Linear Regression Analysis Between Cell Surface Markers and Cartilage Regeneration on Magnetic Resonance Imaging

|  | CD 31 | CD 34 | CD 45 | CD 73 | CD 90 |
| --- | --- | --- | --- | --- | --- |
| 3 months |  |  |  |  |  |
| Mean change of chondral defect area | R^2^ = .005  *P* = .819 | R^2^ = .000  *P* > .999 | R^2^ = .005  *P* = .812 | R^2^ = .232  *P* = .095 | R^2^ = .055  *P* = .440 |
| Ratio of regenerated cartilage area | R^2^ = .059  *P* = .425 | R^2^ = .095  *P* = .305 | R^2^ = .120  *P* = .246 | R^2^ = .126  *P* = .234 | R^2^ = .019  *P* = .655 |
| MOCART score | R^2^ = .117  *P* = .135 | R^2^ = .183  *P* = .144 | R^2^ = .088  *P* = .326 | R^2^ = .010  *P* = .749 | R^2^ = .132  *P* = .223 |
| 6 months |  |  |  |  |  |
| Mean change of chondral defect area | R^2^ = .015  *P* = .691 | R^2^ = .000  *P* = .971 | R^2^ = .028  *P* = .587 | R^2^ = .029  *P* = .577 | R^2^ = .000  *P* = .951 |
| Ratio of regenerated cartilage area | R^2^ = .033  *P* = .554 | R^2^ = .031  *P* = .563 | R^2^ = .005  *P* = .811 | R^2^ = .021  *P* = .638 | R^2^ = .001  *P* = .933 |
| MOCART score | R^2^ = .080  *P* = .350 | R^2^ = .084  *P* = .337 | R^2^ = .014  *P* = .697 | R^2^ = .030  *P* = .572 | R^2^ = .017  *P* = .675 |
| 18 months |  |  |  |  |  |
| Mean change of chondral defect area | R^2^ = .003  *P* = .863 | R^2^ = .001  *P* = .908 | R^2^ = .008  *P* = .776 | R^2^ = .167  *P* = .166 | R^2^ = .008  *P* = .774 |
| Ratio of regenerated cartilage area | R^2^ = .032  *P* = .561 | R^2^ = .023  *P* = .624 | R^2^ = .003  *P* = .849 | R^2^ = .066  *P* = .395 | R^2^ = .007  *P* = .791 |
| MOCART score | R^2^ = .058  *P* = .426 | R^2^ = .071  *P* = .378 | R^2^ = .008  *P* = .775 | R^2^ = .011  *P* = .734 | R^2^ = .000  *P* = .961 |
| 24 months |  |  |  |  |  |
| Mean change of chondral defect area | R^2^ = .007  *P* = .791 | R^2^ = .000  *P* = .944 | R^2^ = .018  *P* = .662 | R^2^ = .171  *P* = .089 | R^2^ = .004  *P* = .833 |
| Ratio of regenerated cartilage area | R^2^ = .044  *P* = .492 | R^2^ = .015  *P* = .693 | R^2^ = .001  *P* = .928 | R^2^ = .231  *P* = .096 | R^2^ = .006  *P* = .800 |
| MOCART score | R^2^ = .030  *P* = .571 | R^2^ = .028  *P* = .586 | R^2^ = .001  *P* = .909 | R^2^ = .062  *P* = .411 | R^2^ = .001  *P* = .940 |

CD, Cluster of Differentiation Antigen; MOCART, magnetic resonance observation of cartilage repair tissue.

Supplementary Table 3. Clinical Follow-up Results per WOMAC scores

|  | ADMSC | *P*-value^†,‡^ | Control | *P*-value^†, ‡^ | *P*-value (ADMSC vs Control)^‡^ |
| --- | --- | --- | --- | --- | --- |
| WOMAC pain |  |  |  |  |  |
| baseline | 8.0 ± 4.0 |  | 6.8 ± 3.6 |  | .908 |
| 3 months | 5.8 ± 3.5 | .169 | 6.0 ± 2.5 | .495 | .908 |
| 6 months | 4.6 ± 3.0 | .088 | 5.4 ± 3.6 | .371 | .908 |
| 18 months | 3.0 ± 2.3 | **.004^*^** | 3.1 ± 2.2 | **.004^*^** | .908 |
| 24 months | 2.9 ± 1.3 | **.004^*^** | 3.8 ± 2.6 | **.024^*^** | .908 |
| WOMAC stiffness |  |  |  |  |  |
| baseline | 4.1 ± 2.1 |  | 2.9 ± 1.9 |  | .303 |
| 3 months | 3.0 ± 2.0 | .101 | 2.8 ± 1.3 | .858 | .683 |
| 6 months | 2.2 ± 1.4 | **.002^*^** | 2.7 ± 1.8 | .858 | .683 |
| 18 months | 1.3 ± 1.1 | **< .001^*^** | 1.5 ± 0.8 | **.016^*^** | .683 |
| 24 months | 1.4 ± 0.8 | .**002^*^** | 2.2 ± 1.5 | .510 | .303 |
| WOMAC function |  |  |  |  |  |
| baseline | 33.8 ± 12.0 |  | 26.2 ± 11.7 |  | .328 |
| 3 months | 26.3 ± 12.5 | .118 | 23.2 ± 9.6 | .409 | .667 |
| 6 months | 22.2 ± 11.5 | .071 | 19.3 ± 13.3 | .113 | .667 |
| 18 months | 13.6 ± 8.0 | **< .001^*^** | 12.3 ± 7.2 | **< .001^*^** | .667 |
| 24 months | 12.8 ± 5.9 | **< .001^*^** | 17.7 ± 9.5 | .062 | .328 |
| WOMAC total |  |  |  |  |  |
| baseline | 45.9 ± 17.1 |  | 35.9 ± 16.1 |  | .343 |
| 3 months | 35.1 ± 16.9 | .113 | 32.0 ± 12.8 | .434 | .811 |
| 6 months | 29.1 ± 15.4 | .061 | 27.4 ± 18.3 | .184 | .811 |
| 18 months | 17.9 ± 10.9 | **< .001^*^** | 16.9 ± 9.7 | **< .001^*^** | .811 |
| 24 months | 17.1 ± 7.6 | **< .001^*^** | 23.7 ± 13.1 | **.049^*^** | .343 |

^α^ Values are present as mean ± standard deviation. ADMSC, adipose-derived mesenchymal stem cell; n.s, not significant; WOMAC, Western Ontario and McMaster Universities Arthritis Index.

^†^ P-value was obtained from paired *t*-test to compare baseline with each postoperative variables.

^‡^ P-value was obtained from Student *t* test and adjusted for multiple comparison using the false discovery rate (FDR).

^*^ Statistical significance was set at < 0.05.

Supplementary Table 4. Clinical Follow-up Results per KOOS scores

|  | ADMSC | *P*-value^†^ | Control | *P*-value^†^ | *P*-value (ADMSC vs Control) ^‡^ |
| --- | --- | --- | --- | --- | --- |
| KOOS pain |  |  |  |  |  |
| baseline | 57.1 ± 21.4 |  | 61.3 ± 17.8 |  | .733 |
| 3months | 64.1 ± 17.5 | .283 | 70.5 ± 13.2 | .125 | .733 |
| 6months | 57.7 ± 13.9 | .195 | 72.0 ± 16.1 | .114 | .733 |
| 18months | 77.1 ± 9.7 | **.020^*^** | 77.6 ± 7.6 | **.004^*^** | .733 |
| 24months | 74.6 ± 9.3 | **.020^*^** | 76.9 ± 12.2 | **.004^*^** | .733 |
| KOOS symptom |  |  |  |  |  |
| baseline | 57.1 ± 23.7 |  | 60.7 ± 13.3 |  | .891 |
| 3 months | 69.0 ± 15.9 | .131 | 67.3 ± 12.9 | .053 | .891 |
| 6 months | 73.9 ± 13.5 | **.021^*^** | 75.3 ± 19.5 | **.017^*^** | .891 |
| 18 months | 84.9 ± 11.5 | **< .001^*^** | 83.8 ± 11.4 | **< .001^*^** | .891 |
| 24 months | 78.0 ± 10.6 | **.016^*^** | 78.6 ± 9.7 | **< .001^*^** | .891 |
| KOOS ADL |  |  |  |  |  |
| baseline | 56.6 ± 16.7 |  | 72.1 ± 12.0 |  | .060 |
| 3 months | 62.9 ± 13.4 | .362 | 70.1 ± 14.1 | .642 | .598 |
| 6 months | 73.0 ± 15.4 | **.045^*^** | 75.6 ±18.3 | .642 | .699 |
| 18 months | 84.3 ± 7.9 | **< .001^*^** | 82.0 ± 11.1 | **.012^*^** | .699 |
| 24 months | 82.1 ± 9.7 | **< .001^*^** | 80.4 ± 12.2 | **.048^*^** | .699 |
| KOOS Sports |  |  |  |  |  |
| baseline | 23.5 ± 15.1 |  | 36.5 ± 21.2 |  | .385 |
| 3 months | 30.0 ± 16.8 | .500 | 33.1 ± 18.7 | .788 | .829 |
| 6 months | 25.8 ± 10.2 | .686 | 33.8 ± 21.4 | .788 | .385 |
| 18 months | 30.0 ± 12.2 | .500 | 29.2 ± 7.9 | .788 | .850 |
| 24 months | 30.0 ± 19.1 | .500 | 38.5 ± 14.1 | .788 | .385 |
| KOOS QoL |  |  |  |  |  |
| baseline | 33.9 ± 19.5 |  | 32.7 ± 12.5 |  | .855 |
| 3 months | 34.6 ± 13.9 | .909 | 38.9 ±12.8 | .217 | .695 |
| 6 months | 41.3 ± 19.7 | .415 | 44.2 ±13.4 | **.021^*^** | .833 |
| 18 months | 52.9 ± 17.0 | **.004^*^** | 47.6 ±12.1 | **.008^*^** | .695 |
| 24 months | 48.1 ± 10.9 | **.020^*^** | 52.8 ±17.3 | **.008^*^** | .695 |

^α^ Values are present as mean ± standard deviation. ADL, activities of daily living; ADMSC, adipose-derived mesenchymal stem cell; KOOS, Knee Injury and Osteoarthritis Outcome Score; n.s, not significant; QoL, quality of life.

^†^ P-value was obtained from paired *t*-test to compare baseline with each postoperative variables.

^‡^ P-value was obtained from Student *t* test and adjusted for multiple comparison using the false discovery rate (FDR).

^*^ Statistical significance was set at < 0.05.

Supplementary Table 5. Comparison of mean improvement from baseline in WOMAC and KOOS scores.

|  | ADMSC | Control | *P*-value (ADMSC vs Control) ^†^ |
| --- | --- | --- | --- |
| WOMAC pain |  |  |  |
| 3 months | 2.2 ± 5.4 | 0.8 ± 4.2 | .482 |
| 6 months | 3.4 ± 6.0 | 1.4 ± 4.6 | .482 |
| 18 months | 5.0 ± 3.9 | 3.7 ± 3.2 | .482 |
| 24 months | 5.1 ± 4.6 | 3.0 ± 3.6 | .482 |
| WOMAC stiffness |  |  |  |
| 3 months | 1.1 ± 2.2 | 0.1 ± 2.0 | .247 |
| 6 months | 1.9 ± 2.4 | 0.1 ± 2.1 | **.076** |
| 18 months | 2.8 ± 1.7 | 1.4 ± 1.4 | **.054** |
| 24 months | 2.7 ± 2.2 | 0.7 ± 2.0 | **.054** |
| WOMAC function |  |  |  |
| 3 months | 7.5 ± 16.1 | 3.0 ± 12.6 | .484 |
| 6 months | 11.5 ± 19.3 | 6.9 ± 13.3 | .484 |
| 18 months | 20.2 ± 12.2 | 13.9 ± 9.4 | .310 |
| 24 months | 20.9 ± 13.4 | 8.5 ± 12.6 | **.088** |
| WOMAC total |  |  |  |
| 3 months | 10.8 ± 22.8 | 4.0 ± 17.4 | .592 |
| 6 months | 16.8 ± 27.2 | 8.5 ± 19.3 | .506 |
| 18 months | 28.0 ± 16.7 | 19.0 ± 12.6 | .224 |
| 24 months | 28.8 ± 19.4 | 12.2 ± 17.2 | **.080** |
| KOOS pain |  |  |  |
| 3months | 7.1 ± 22.6 | 9.2 ± 20.1 | >.999 |
| 6months | 10.7 ± 24.8 | 10.7 ± 20.6 | >.999 |
| 18months | 20.1 ± 21.0 | 16.2 ± 14.8 | >.999 |
| 24months | 17.5 ± 20.5 | 15.6 ± 12.9 | >.999 |
| KOOS symptom |  |  |  |
| 3 months | 11.8 ± 26.3 | 6.6 ± 11.1 | .806 |
| 6 months | 16.5 ± 21.3 | 14.6 ± 18.1 | .806 |
| 18 months | 27.7 ± 20.9 | 23.1 ± 10.4 | .806 |
| 24 months | 20.8 ± 23.6 | 17.9 ± 13.6 | .806 |
| KOOS ADL |  |  |  |
| 3 months | 6.3 ± 24.1 | -2.0 ± 14.9 | .301 |
| 6 months | 16.4 ± 24.6 | 3.5 ± 19.4 | .200 |
| 18 months | 27.7 ± 17.0 | 9.9 ± 9.7 | .**012^*^** |
| 24 months | 25.6 ± 16.9 | 8.3 ± 11.9 | .**012^*^** |
| KOOS Sports |  |  |  |
| 3 months | 6.5 ± 25.6 | -3.5 ± 28.8 | .622 |
| 6 months | 2.3 ± 18.2 | -2.7 ± 16.7 | .622 |
| 18 months | 6.5 ± 17.8 | -7.3 ± 23.9 | .428 |
| 24 months | 6.5 ± 21.8 | 2.0 ± 25.2 | .622 |
| KOOS QoL |  |  |  |
| 3 months | 0.7 ± 22.8 | 6.3 ± 17.3 | .622 |
| 6 months | 7.5 ± 25.4 | 11.5 ± 14.8 | .622 |
| 18 months | 19.0 ± 16.2 | 14.9 ± 15.2 | .622 |
| 24 months | 14.2 ± 16.7 | 20.1 ± 20.0 | .622 |

^α^ Values are present as mean ± standard deviation. ADL, activities of daily living; ADMSC, adipose-derived mesenchymal stem cell; KOOS, Knee Injury and Osteoarthritis Outcome Score; n.s, not significant; QoL, quality of life; WOMAC, Western Ontario and McMaster Universities Arthritis Index.

^†^ P-value was obtained from Student *t* test and adjusted for multiple comparison using the false discovery rate (FDR).

^*^ Statistical significance was set at < 0.05.

Supplementary table 6. Biomarker Evaluation at postoperative 24 months^α^

|  | ADMSC  (n = 13) | Control  (n = 13) | *P* value |
| --- | --- | --- | --- |
| Serum biomarkers |  |  |  |
| COMP, µg/ml | 2.6 ± 1.3 | 2.5 ± 1.2 | .72^b^ |
| CTX-I, ng/ml | 0.40 ± 0.16 | 0.35 ± 0.14 | .31^b^ |
| CTX-II, ng/ml | 6.5 ± 1.6 | 7.1 ± 2.3 | .36^b^ |
| IL-10, pg/ml | 3.3 ± 0.5 | 4.0 ± 1.4 | .10^b^ |
| TSG-6, units/ml | 11.2 ± 4.2 | 12.1 ± 3.7 | .51^b^ |
| Urine biomarker |  |  |  |
| Urine CTX-II, ng/mmol | 1032.3 ± 1968.5 | 1041.4 ± 1623.8 | >.99^b^ |
| Synovial biomarker |  |  |  |
| TSP-2, ng/ml | 3.7 ± 1.7 | 1.5 ± 1.9 | .09^b^ |

^α^ Values are presented as mean ± standard deviation.

ADMSC, adipose-derived mesenchymal stem cell; COMP, cartilage oligomeric matrix protein; CTX-I, C-terminal telopeptide of collagen type-I; CTX-II, C-terminal telopeptide of collagen type-II; IL-10, interleukin-10; TSG-6, tumor necrosis factor-inducible gene-6; TSP-2, thrombospodin-2.

^a^ Student *t* test

^b^ Mann-Whitney *U* test

^*^ Statistical significance was set al *P*-value < 0.05

Supplementary Table 7. The Number of patients experiencing adverse events at the System Organ Class level

|  | **ADMSC**  (n=13) | **Control**  (n=13) |
| --- | --- | --- |
| **System Organ Class** | 13 | 21 |
| Cardiac disorders | 0 | 2 |
| Gastrointestinal disorders | 1 | 2 |
| General disorders and administration site conditions | 0 | 0 |
| Hepatobiliary disorder | 1 | 0 |
| Musculoskeletal and connective tissue disorders | 8 | 7 |
| Nervous system disorders | 1 | 0 |
| Reproductive system and breast disorder | 0 | 4 |
| Respiratory, thoracic and mediastinal disorder | 1 | 1 |
| Skin and subcutaneous tissue disorder | 1 | 5 |
| **Preferred Term** |  |  |
| Allergic reaction | 0 | 1 |
| Appendicitis | 0 | 1 |
| Backache | 3 | 2 |
| Breast pain | 0 | 1 |
| Chest pain | 0 | 1 |
| Climacteric syndrome | 0 | 1 |
| Constipation | 0 | 1 |
| Dermatitis | 1 | 3 |
| Donor-site pain^†^ | 0 | - |
| Dizziness | 1 | 0 |
| Dysuria | 0 | 1 |
| Endometrial polyp | 0 | 1 |
| Fatty liver | 1 | 0 |
| Generalized hyperhidrosis | 0 | 1 |
| Hypertension | 0 | 1 |
| Musculoskeletal pain | 5 | 3 |
| Nasopharyngitis | 1 | 1 |
| Nausea | 1 | 0 |
| Sciatica | 0 | 2 |

^†^ Persistent donor-site pain despite of taking pain medicine.

Supplementary Figure 1.


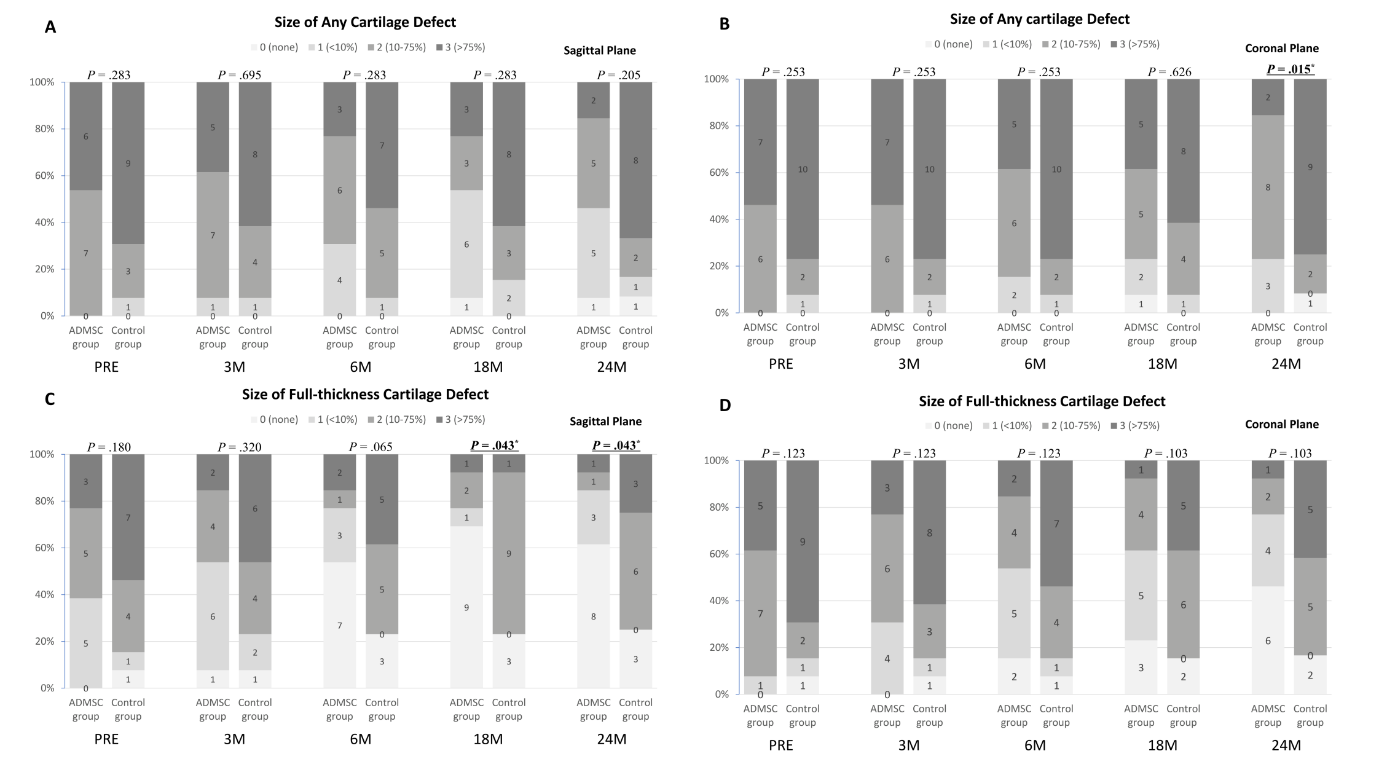


Supplementary figure 1. MOAK grade of cartilage defect in the medial compartment based on preoperative and postoperative serial MRI evaluations. The size of any articular cartilage defect is shown in sagittal (A) and coronal (B) planes. The size of full-thickness articular cartilage defect is shown in sagittal (C) and coronal (D) planes. The MOAK grade of ADMSC group showed smaller in the size of any articular cartilage defect than those of control group at postoperative 24 months in coronal planes. Moreover, the MOAK grade of ADMSC group showed significantly smaller in the size of full-thickness articular cartilage defect in the MFC than those of control group at postoperative 18 and 24 months in sagittal plane. MRI, magnetic resonance imaging; MOAK, MRI Osteoarthritis Knee Score; ADMSC, adipose-derived mesenchymal stem cell.
